# Comparison of test performance of a conventional PCR and two field-friendly tests to detect *Coxiella burnetii* DNA in ticks using Bayesian latent class analysis

**DOI:** 10.3389/fvets.2024.1396714

**Published:** 2024-06-19

**Authors:** Maureen W. Kamau, Carmel Witte, Wynand Goosen, Mathew Mutinda, Jandouwe Villinger, Dennis Getange, Rua Khogali, Michael E. von Fricken, Eric Maurice Fèvre, Dawn Zimmerman, Yvonne-Marie Linton, Michele Miller

**Affiliations:** ^1^Mpala Research Centre, Nanyuki, Kenya; ^2^Division of Molecular Biology and Human Genetics, Department of Science, and Innovation – National Research Foundation Centre of Excellence for Biomedical Tuberculosis Research, Faculty of Medicine and Health Sciences, South African Medical Research Council Centre for Tuberculosis Research, Stellenbosch University, Stellenbosch, South Africa; ^3^Global Health Program, Smithsonian National Zoo Conservation Biology Institute, Washington, DC, United States; ^4^The Center for Wildlife Studies, South Freeport, ME, United States; ^5^Kenya Wildlife Service, Nairobi, Kenya; ^6^International Centre of Insect Physiology and Ecology (ICIPE), Nairobi, Kenya; ^7^College of Public Health and Health Professionals, Department of Environmental and Global Health University of Florida, Gainesville, FL, United States; ^8^International Livestock Research Institute, Nairobi, Kenya; ^9^Institute of Infection, Veterinary and Ecological Sciences, University of Liverpool, Liverpool, United Kingdom; ^10^Veterinary Initiative for Endangered Wildlife, Bozeman, MT, United States; ^11^Walter Reed Biosystematics Unit (WRBU) Smithsonian Institution Museum Support Center, Suitland, MD, United States; ^12^One Health Branch, Walter Reed Army Institute of Research (WRAIR), Silver Spring, MD, United States; ^13^Department of Entomology, Smithsonian Institution, National Museum of Natural History (NMNH), Washington, DC, United States

**Keywords:** *Coxiella burnetii*, diagnostics, Q fever, sensitivity, specificity, ticks, wildlife

## Abstract

**Introduction:**

*Coxiella burnetii* (*C. burnetii*)-infected livestock and wildlife have been epidemiologically linked to human Q fever outbreaks. Despite this growing zoonotic threat, knowledge of coxiellosis in wild animals remains limited, and studies to understand their epidemiologic role are needed. In *C. burnetii*-endemic areas, ticks have been reported to harbor and spread *C. burnetii* and may serve as indicators of risk of infection in wild animal habitats. Therefore, the aim of this study was to compare molecular techniques for detecting *C. burnetii* DNA in ticks.

**Methods:**

In total, 169 ticks from wild animals and cattle in wildlife conservancies in northern Kenya were screened for *C. burnetii* DNA using a conventional PCR (cPCR) and two field-friendly techniques: Biomeme’s *C. burnetii* qPCR Go-strips (Biomeme) and a new *C. burnetii* PCR high-resolution melt (PCR-HRM) analysis assay. Results were evaluated, in the absence of a gold standard test, using Bayesian latent class analysis (BLCA) to characterize the proportion of *C. burnetii* positive ticks and estimate sensitivity (Se) and specificity (Sp) of the three tests.

**Results:**

The final BLCA model included main effects and estimated that PCR-HRM had the highest Se (86%; 95% credible interval: 56–99%), followed by the Biomeme (Se = 57%; 95% credible interval: 34–90%), with the estimated Se of the cPCR being the lowest (24%, 95% credible interval: 10–47%). Specificity estimates for all three assays ranged from 94 to 98%. Based on the model, an estimated 16% of ticks had *C. burnetii* DNA present.

**Discussion:**

These results reflect the endemicity of *C. burnetii* in northern Kenya and show the promise of the PCR-HRM assay for *C. burnetii* surveillance in ticks. Further studies using ticks and wild animal samples will enhance understanding of the epidemiological role of ticks in Q fever.

## 1 Introduction

*Coxiella burnetii* (*C. burnetii*) is a small gram-negative bacterium and the etiological agent of Q fever, a zoonotic infection with distribution throughout most of the world (1). Although rarely fatal in humans, the disease may lead to substantial morbidity and can be highly debilitating, even with treatment (2). Infections also result in significant economic losses associated with control and treatment of the disease in domestic animals as well as production losses resulting from poor reproductive performance (3). Human Q fever cases are commonly associated with livestock contact, such as the notable Q fever epidemic of more than 4,000 cases in The Netherlands during 2007–2010 (1) that originated from a *C. burnetii* strain in goats (4, 5). However, an increasing number of global reports link human Q fever cases to contact with wild animals (6). For instance, a capybara (7), and three-toed sloth (*Bradypus tridactylus*) (8) were epidemiologically linked to community-acquired pneumonia caused by *C. burnetii* infection in French Guiana (7–10). In another study, two cases of Q fever in South Wales, Australia, were linked to handling kangaroo joeys, and mowing lawns contaminated with kangaroo feces (11). Clinical disease associated with *C. burnetii* infection has also been reported in wild animals, including critically endangered species such as the dama gazelle (*Nanger dama mhorr*) and saiga antelope (*Saiga tatarica tatarica*) (12, 13). Knowledge of the clinical outcome of coxiellosis in wildlife is still limited, however placentitis and reproductive failure have been reported, raising concerns regarding the impact of *C. burnetii* on conservation breeding programs (14). Despite the increasing importance of coxiellosis in wild animals and the recognized risk to human health, study of the disease in wild animals and their environment has been largely neglected (6).

In Kenya, Q fever is ranked among the top 10 priority zoonotic diseases (15). A recent systematic review suggested that both human and animal *C. burnetii* infections are largely unrecognized and therefore underestimated due to limited diagnostic capacity at remote satellite laboratories (16). The review also highlighted that epidemiologic studies in high-risk regions, where there is increased livestock-wildlife interactions, are required to improve our understanding of the role that wild animals play in Q fever outbreaks (16). Previously, studies on Q fever in Kenya have focused on livestock (17–22), humans (23–25), or both (26–28), while only a few studies addressed *C. burnetii* infections in wild animals and their environment (29, 30). Active surveillance programs for wild animal diseases are expensive, logistically difficult, and may result in limited sample sizes, which could underestimate the abundance of *C. burnetii* (14, 31).

An alternative approach to surveillance is to screen disease vectors, such as ticks, to estimate *C. burnetii* abundance in an ecosystem (32). Ticks are ecological bridges for *C. burnetii* spread between wild animals and domestic animals (2). Ticks can get infected with *C. burnetii* when feeding on a bacteremic host and transmit the bacterium to a different host during subsequent feeding (33). Even if the ticks only acquire the bacterium without transmitting it, pathogen surveillance using ticks could provide insights into the presence and abundance of *C. burnetii* in wild animal ecosystems (34). Ticks are easy to collect and can be sampled to represent widespread geographical coverage. In known *C. burnetii* endemic areas, such as northern Kenya (35), tick species collected from wild animals and their environments have been found to harbor *C. burnetii* (20, 29, 36, 37). Furthermore, in these same regions, there was a strong correlation between *C. burnetii* seropositivity in livestock and their infestation with *C. burnetii* infected ticks (20, 21, 26). Therefore, detection of *C. burnetii* in ticks offers a promising approach to further *Coxiella* surveillance in wild animals and their ecosystems.

Therefore, the aim of this study was to compare the performance of three tests for screening tick vectors for *C. burnetii*. We compared (1) conventional PCR assay (cPCR), against two field-friendly techniques: (2) the *C. burnetii* qPCR Go-strips test (Biomeme, Philadelphia, Pennsylvania, United States) on the Biomeme Franklin™ three 9 platform (referred to here as “the Biomeme”) and (3) a *C. burnetii* PCR high-resolution melt (PCR-HRM) assay. Additionally, the proportion of ticks with *C. burnetii* DNA was used as an indicator of pathogen abundance in the ecosystem. Because a gold standard test is not available, the performance of the three assays were compared using a Bayesian latent class analysis (BLCA) to assess the feasibility of using ticks for surveillance of *C. burnetii* in an ecosystem.

## 2 Materials and methods

### 2.1 Tick collection, identification, and DNA extraction

This study utilized DNA from ticks whose collection and processing has been previously described (38); our study design was retrospective and data collection was opportunistic and occurred before *C. burnetti* DNA testing was performed. Ticks were opportunistically collected (convenience sampling) from 179 wild animals and cattle during routine veterinary interventions in wildlife conservancies within Nyeri, Meru, Laikipia, Isiolo, and Marsabit counties (northern Kenya), between May 2011 and April 2019. The ticks were collected from 13 different wild animal species, as well as cattle present in the conservancies. Wildlife species included: black rhinoceros (*Diceros bicornis*), African buffalo (*Syncerus caffer*), African elephant (*Loxodonta africana*), giraffe (*Giraffa camelopardalis*), Grévy’s zebra (*Equus grevyi*), hartebeest (*Alcelaphus buselaphus*), impala (*Aepyceros melampus*), leopard (*Panthera pardus*), lion (*Panthera leo*), plains zebra (*Equus quagga*), spotted hyena (*Crocuta crocuta*), white rhinoceros (*Ceratotherium simum*), and African wild dog (*Lycaon pictus*), as well as farmed Boran and zebu cattle (*Bos indicus*). Cattle and wild animals were visually examined for ticks during clinical interventions and, if detected, several ticks were collected from each animal and placed in a tube containing 70% ethanol for preservation of the tick DNA. Collection location, date, and host species were recorded on each tube. Tick samples were stored in a −80°C freezer until analyzed.

Field immobilizations and wild animal sample collections were conducted according to Kenya Wildlife Service standard operating procedures. No animals were specifically immobilized or handled for this study. This study was approved by the Stellenbosch University Animal Use and Care (ACU-2023-27912) and Kenya’s National Commission for Science, Technology, and Innovation (NACOSTI/P/21/8054).

For *C. burnetii* testing, one tick per host animal (i.e., 179 ticks total, representing 179 different individual animals) was selected. A subset of these were identified to the genus level based on metagenomics, as previously described (38). Each tick (*n* = 179) was placed into liquid nitrogen then homogenized in 1.5 mL microfuge tubes, containing 200 μL of 1x phosphate-buffered saline (PBS), and 150 mg of 0.1 mm and 750 mg of 2.0 mm yttria-stabilized zirconium (YSZ) oxide beads (Glen Mills, Clifton, New Jersey, United States), using a Mini-Beadbeater-16 (BioSpec, Bartlesville, Oklahoma, United States) for 1 min. Tick DNA was extracted with the Qiagen DNeasy Blood and Tissue Kit (Qiagen, Germantown, Maryland, United States) using 100 μL of the tick homogenate, according to manufacturer’s instructions. Extracted DNA was stored at −80°C prior to PCR testing. Prior to performing the PCRs, DNA quantity and quality were measured using the Qubit dsDNA High Sensitivity Assay kit (Thermofisher Scientific, Waltham, Massachusetts, United States) and the Qubit 4 fluorometer (Thermofisher Scientific), as described by the manufacturer.

### 2.2 *Coxiella* PCR detection

Each of the tick DNA samples were screened for presence of *C. burnetii* DNA using three separate methods: cPCR, PCR-HRM, and the Biomeme’s *C. burnetii* qPCR Go-strips (Biomeme). The PCR-HRM and cPCR assays were performed at the Molecular Biology and Bioinformatics Unit at the International Centre for Insect Physiology and Ecology (ICIPE, Nairobi, Kenya). Tests using the Biomeme were conducted at Mpala Research Centre (Laikipia, Kenya), following manufacturers’ instructions. Primers utilized in all PCRs targeted the IS*1111* transposase elements of the *C. burnetii* genome (Table 1).

**Table 1:** Test type, primer sequence and criteria for positive results for the three evaluated *Coxiella burnetii* PCR tests targeting the IS*1111* transposase elements in the *C. burnetii* genome, using DNA from ticks collected from wildlife and cattle in Kenya between May 2011 and 2019.

Test	Primer name	Primer sequence (5′-3′)	Amplicon length (bp)	Positive result	References
Conventional PCR	Trans 1	TATGTATCCACCGTAGCCAGTC	687	Samples that have a clear band at the expected amplicon size of 687 bp	Hoover et al. (39)
	Trans 2	CCCAACAACACCTCCTTATTC			
Biomeme qPCR	Proprietary	Proprietary *C. burnetii* qPCR Go-strips test (Biomeme, Philadelphia, Pennsylvania, United States)	290	Samples that had a Cq* value ≤40	N/A
PCR-HRM	*C_burnetii*_HRM-F	GGAACTTGTCAGAGATGATTTGGT	150	Samples whose melt profiles’ peaks and shape was similar to the positive control	This study
	*C_burnetii*_HRM-R	AGAGTTCCCGACTTGACTCG			

*Cq, quantification cycle value.

#### 2.2.1 *Coxiella burnetii* conventional PCR

This PCR assay included a touch-down PCR amplification, followed by 1.5% agarose gel electrophoresis to visualize the PCR product. Primers used were trans 1 and trans 2 (Table 1), which amplified a 687 bp fragment of the repetitive, transposon-like element, as described previously (40).

The 11 μL PCR mixture consisted of 0.5 μL of each forward and reverse primer at a final reaction concentration of 0.5 μM, 2 μL of template DNA, 2 μL of HOT FIREPol^®^ Blend Master Mix (Solis BioDyne, Tartu, Estonia), and 6 μL of nuclease free water. Known *C. burnetii* positive tick DNA samples and nuclease free water were included on each plate as a positive control and no template control, respectively. The Supercycler Trinity thermocycler (Kyratec, Queensland, Australia) was used for the PCR. The cycling conditions included an initial enzyme activation step at 95°C for 12 min. This was followed by 5 cycles of denaturation at 95°C for 30 s, an annealing step where the temperature was lowered by 1°C between successive steps from 66°C to 61°C for 45 s, and extension at 72°C for 1 min. An additional 35 cycles consisting of denaturation at 95°C for 30 s, annealing at 61°C for 30 s, extension at 72°C for 45 s, and a final extension step at 72°C for 7 min were added. The PCR products were stained with ethidium bromide and loaded onto a 1.5% agarose gel submerged in a TAE (40 mM Tris-acetate, 1 mM EDTA) buffer-filled electrophoresis chamber set at 97 volts for 42 min. The sizes of the PCR products were determined using an O’rangeRuler 100 bp DNA ladder (Thermofisher Scientific). Images of the gels were recorded.

Positive samples had a clear 687 bp band and were reamplified using trans 1 and trans 2 primers with cycling conditions as described in the previous paragraph (40). The amplicons were then cleaned with the ExoSAP-IT PCR Product Cleanup kit (Affymetrix, Santa Clara, California, United States), according to manufacturer’s instructions. The purified amplicons were submitted for Sanger sequencing at Macrogen (Amsterdam, The Netherlands) to confirm the identity of the amplified sequences.

#### 2.2.2 Biomeme *Coxiella burnetii* Go-strip qPCR assay

The *C. burnetii* Go-strips (Biomeme) were supplied as individually packaged three-well qPCR test strips. Each three-well qPCR test strip contained lyophilized master mix, primers, and probes. The primers used for this assay target a 290 bp fragment of the IS*1111* transposase elements in the genome of *C. burnetii* (Sarah Senula, pers. comm., February 8, 2022).

The Biomeme *C. burnetii* Go-strip qPCR assays were performed according to the manufacturer’s instructions. In brief, 20 μL of tick DNA eluent was transferred into a single well of the Biomeme Go-Strip and a positive control included for each batch of samples. Biomeme provided a synthetic *C. burnetii* positive control on Whatman paper punches. Each punch was resuspended by adding 400 μL of TAE buffer to the punch and incubating overnight at room temperature. A 20 μL aliquot of the positive control eluent represented 5 copies per reaction. The Go-Strips were loaded into the Biomeme Franklin^™^ three9 Real-Time PCR Thermocycler and the Biomeme Go application was launched on the connected Android phone (Samsung s8+, Samsung, Ridgefield Park, New Jersey, United States). Using the Biomeme Go application, each sample was individually labeled with a sample identifier, and the QR code on the *C. burnetii* Go strips pouch scanned to launch the Biomeme *C. burnetii* qPCR cycling parameters. The PCR cycle consisted of an initial denaturation step for 1 min at 95°C, followed by 45 cycles for 1 s at 95°C, and annealing for 20 s at 60°C. The Biomeme Go mobile application provided views of amplification plots of raw data in real time. *S*amples positive for *C. burnetii* DNA showed fluorescence on the green channel and the Cq value per sample was also recorded. Samples with a Cq value ≤ 40 were categorized as positive.

#### 2.2.3 *Coxiella burnetii* PCR-HRM analysis

The PCR-HRM assay involved a PCR step followed by HRM analysis, using *C_burnetii_*HRM-F and *C_burnetii*_HRM-R primers, designed using Geneious Prime software (Biomatters, Inc., San Diego, California, United States) to cross sequences in which *Coxiella*-endosymbiont sequences have insertions and deletions, thus preventing amplification of *Coxiella*-endosymbionts. The amplification regions were identified through alignments of nucleotide sequences available in the GenBank database, ensuring that the 3′ regions of the primers were specific to *C. burnetii* and not the closely related *Coxiella* endosymbionts (Supplementary Figure S1). The specificity of each primer set was evaluated using the Basic Local Alignment Search Tool (BLAST) from the National Center for Biotechnology Information (NCBI) database. The primer sets were adjusted to achieve optimal annealing temperatures and amplification efficiency. Specificity of the designed primers were tested using samples confirmed to be positive for *C. burnetii, Coxiella* endosymbionts, and nuclease free water, as a negative control.

The PCRs were conducted in 11 μL reaction volumes consisting of 2 μL of HOTFIREPol EvaGreen HRM mix (Solis BioDyne), 0.5 μL of each forward and reverse primer at a final reaction concentration of 0.5 μM, 2 μL of template DNA, and 6 μL of nuclease free water. Known *C. burnetii* positive tick DNA samples and nuclease free water were included in each test batch as a positive control and no template control, respectively. A HRM-capable MIC-4 thermocycler (Bio Molecular Systems, Upper Coomera, Queensland, Australia) was used for DNA amplification. The PCR cycling conditions included an initial enzyme activation step at 95°C for 12 min, followed by 40 cycles of denaturation at 95°C for 30 s, annealing at 62°C for 25 s, extension at 72°C for 20 s, and a final extension at 72°C for 7 min. The PCR was immediately followed by the melt curve analysis during which amplicons were gradually melted at 0.1°C increments from 75°C to 95°C, with fluorescence acquisition every 0.5 s. The Mic qPCR Software automatically generated raw graphs of fluorescence against temperature (°C) from the fluorescence acquisition data. The samples’ melt profiles were compared to that of the positive control, based on their melt-curve peaks and curve shapes. Positive samples showed melt profiles with peaks and shapes that aligned with the positive control. To confirm HRM based species identification, the positive samples were reamplified by conventional PCR using COX-F (GTCTTAAGGTGGGCTGCGTG) and COX-R (CCCCGAATCTCATTGATCAGC) primers, targeting a 295 bp fragment of the IS*1111* transposase elements in the *C. burnetii* genome (41). The cycling parameters were programed as previously described in the conventional PCR section. The PCR products were visualized as described above. All samples with 295 bp bands were considered positive, then purified with the ExoSAP-IT PCR Product Cleanup kit (Affymetrix) and submitted for Sanger sequencing at Macrogen to confirm the identity of the amplified sequences.

### 2.3 Data and statistical analyses

Chromatogram files were imported into Geneious Prime software version 2022 (Biomatters Inc.) for trimming, editing, and alignment. To generate consensus sequences, the study sequences were queried against known sequences in GenBank using the Basic Local Alignment Search Tool to reveal their identity and relation to currently deposited sequences (42). The study sequences were then aligned to all available *C. burnetii* sequences in GenBank using the MAFFT plugin (Biomatters Inc.) in Geneious Prime software (Biomatters Inc., version 2022).

The numbers of *C. burnetii* DNA positive and negative ticks were summarized for each method and positive results were reported as proportions of the total number of samples tested. The number of positive and negative results were also summarized by tick genus and host species. Agreement between the three possible pairs of PCR tests was calculated with Cohen’s Kappa coefficient using the psych package (43) in R statistical software (44). A Kappa coefficient close to 1 indicated very good agreement, while 0 indicated poor agreement between tests (45).

#### 2.3.1 Bayesian latent class analysis

A Bayesian latent class analysis (BLCA) was used to estimate the proportion of ticks with *C. burnetii* DNA and evaluate the performance of the three tests in the absence of a gold standard reference test. This analytic approach is well-established in human medicine and veterinary science (46–48) and the World Organization for Animal Health has accepted this method for diagnostic test validation (49). A checklist for BLCA reporting standards (47) is included in Supplementary Appendix S2.

For the present study, a three-test, one population model was used with the latent class defined as whether a tick is positive or negative for *C. burnetii* DNA, as an indicator of *C. burnetii* presence and abundance in the ecosystem. This is a similar approach as previous studies that have shown density estimates of infected ticks are better predictors of the likelihood of Lyme disease occurrence than pathogen or tick abundance alone (50). The model used in the current study included binary test outcomes from the cPCR, Biomeme Go-Strips, and PCR-HRM assays. Observations from each tick were included in the analysis if results were available for all three tests. The model was structured assuming that the different combinations of positive and negative test results followed a multinomial distribution. The analysis was implemented in OpenBugs within the R statistical software (51) and RStudio environments (52) using packages r2OpenBugs (53). Programming code for the model implementation was adapted from Cheung et al. (48) and Salgadu et al. (54) and is provided in Supplementary Appendix S3. The model was fit using non-informative priors for Bayesian estimates of Se and Sp due to either limited data (cPCR) or no published data (Biomeme and PCR-HRM) on *C. burnetii* detection in African ticks.

Bayesian estimates of the proportion of ticks with *C. burnetii* DNA, Se, and Sp were generated using a Markov Chain Monte Carlo algorithm. A model was fit to the data that assumed all tests were independent. While all three PCR tests are based on the detection of the IS*1111* transposase elements in the *C. burnetii* genome, the primers used anneal and amplify different segments and lengths of the IS*1111* transposase elements. Therefore, these were considered independent detection methods. Three separate chains were run for 30,000 iterations, with the first 10,000 discarded as “burn-in” based on examination of trace plots. Model convergence and unimodality were assessed by visual inspection of the trace plots, autocorrelation plots, and posterior median distributions of each parameter with the R packages “coda” (55) and “mcmplots” (56). Posterior median estimates of each parameter and their corresponding 95% credible intervals were reported.

## 3 Results

Ticks were identified to the genus level in 67 out of 179 samples; 112 ticks were not assigned a genus since metagenomic data were unavailable. The majority of identified ticks were *Rhipicephalus* spp. (64/67), and 3/67 of the ticks belonged to the *Amblyomma* genus. Extracted DNA quantity from ticks ranged between 0.03 and 116 ng/μL. Samples from 179 ticks were tested by conventional PCR and the PCR-HRM techniques, while 169 samples were tested on the Biomeme (due to limited availability of kits). Summaries of test results by source host species and tick genera are presented in Supplementary Table S1. In total, 9/179 (5.0%) ticks tested positive for *C. burnetii* DNA by cPCR, 21/169 (12.4%) tested positive with the Biomeme PCR, and 32/179 (17.9%) tested positive by PCR-HRM. Frequency distributions of positive test combinations are shown in Table 2. A figure showing the melt profiles of samples used to test the specificity of the *C_burnetii_*HRM-F and *C_burnetii*_HRM-R primers is included in Supplementary Figure S2.

**Table 2:** Results for 169 tick DNA samples tested for *Coxiella burnetii* by conventional PCR, Biomeme’s commercially available *C. burnetii* Go-Strip assay, and a *C. burnetii* PCR-high resolution melt (HRM) technique.

Conventional PCR	Biomeme	PCR-HRM	Total samples with test result combination (%)
Positive	Positive	Positive	3 (1.8%)
Positive	Positive	Negative	0
Positive	Negative	Positive	3 (1.8%)
Negative	Positive	Positive	11 (6.5%)
Positive	Negative	Negative	3 (1.8%)
Negative	Positive	Negative	7 (4.1%)
Negative	Negative	Positive	14 (8.3%)
Negative	Negative	Negative	128 (75.7%)
Total			169

There was moderate agreement beyond what would be expected by chance between the PCR-HRM and the Biomeme test results (Kappa = 0.46; 95% CI: 0.28–0.64), while agreement was fair between the cPCR and PCR-HRM results (Kappa = 0.23; 95% CI: 0.053–0.41) and only slight agreement between the cPCR and Biomeme results (Kappa = 0.14; 95% CI: 0.061–0.33).

The BLCA model was fit to the 169 data points for tick samples with a test result for all three assays. Posterior median estimates and 95% credibility intervals of Se, Sp, and the proportion of ticks with *C. burnetii* DNA are shown in Table 3. The model estimated that the PCR-HRM assay had the highest Se (86%; 95% credible interval: 56–99%) followed by the Biomeme (Se =57%; 95% credible interval: 34–90%), and the lowest Se for the cPCR (24, 95% credible interval: 10–47%). Specificity estimates ranged from 94 to 98% for all three assays. The estimated proportion of ticks with *C. burnetii* DNA was 16% (95% credible interval: 8–28%).

**Table 3:** Results from the Bayesian latent class model estimating the sensitivity and specificity of three assays (conventional PCR, Biomeme’s commercially available *C. burnetii* Go-Strip Assay, and a *C. burnetii* PCR-HRM technique) and the estimated proportion of ticks carrying *C. burnetii*.

Parameter	Posterior median estimate (%)*	95% credibility interval
**Sensitivity**		
Conventional PCR	24%	10–47%
Biomeme	57%	34–90%
PCRHRM	86%	56–99%
**Specificity**		
Conventional PCR	98%	94–100%
Biomeme	96%	91–100%
PCRHRM	94%	86–100%
Proportion with *C. burnetti*	16%	0.08–28%

*The model assumes independence across assays. Model estimates were generated from 169 tick samples collected from wildlife and cattle in Kenya between May 2011 and 2019. The Deviance Information Criterion (DIC) of the final model was 27.91 (95% credibility interval: 23.41–37.26%).

## 4 Discussion

The pathogen *C. burnetii* remains endemic in rural regions of northern Kenya, posing a risk to both human and animal health (35). Although ticks have been shown to play a role in *C. burnetii* transmission, their use for non-invasive pathogen surveillance in these ecosystems, shared by livestock, wildlife, and humans, has been largely neglected due to limited diagnostic capability at remote satellite laboratories (2, 16, 57). Therefore, the goal in the present study was to fill this gap by using BLCA to compare cPCR, the Biomeme, and PCR-HRM assays, using tick DNA, to provide information on detection methods, presence and abundance of *C. burnetii* in wild animal conservancies in northern Kenya. The study found that all three assays detected *C. burnetii* DNA in whole tick DNA extracts, although a higher Se was found with the PCR-HRM and Biomeme assays at 86, and 57%, respectively, compared to the cPCR (24%). All three assays had high Sp (94–98%), therefore, the assays had a high probability of identifying truly negative ticks. Using BLCA, the estimated proportion of ticks that were positive was 16%, which demonstrated the feasibility of detecting *C. burnetii* using non-invasively collected samples for ecosystem surveillance and infection risk evaluation.

All three PCR methods detected *C. burnetii* positive ticks, however, the Se of the assays varied. The PCR-HRM assay had the highest Se, estimated at 86%, which supports its selection as a screening test for identifying *C. burnetii* positive ticks. Although the Biomeme assay had a lower Se of 57%, it might be a more convenient method for screening in remote areas. The currently used cPCR assay had a low Se (24%) when used with tick samples, which could be attributed to differences between cPCR and qPCR assays (58). In general, cPCR assays use longer amplicons and are qualitative, whereas qPCR assays are quantitative and use shorter amplicons, which ensure higher PCR efficiency and improved sensitivity (58, 59).

While PCR-HRM was found to be more sensitive, both the Biomeme and PCR-HRM had sensitivities and specificities higher than the currently used cPCR. Both the Biomeme and PCR-HRM platforms use portable, battery charged thermocyclers, making them field deployable and more reliable for remote satellite laboratories where power supply may be unreliable. At the time of this report, the Biomeme Franklin thermocycler and the MIC-4 thermocycler were similarly priced; however, the running cost for the Biomeme was approximately tenfold higher in our setting, compared to running the PCR-HRM using the MIC-4 thermocycler. Costs and availability will vary depending on setting and likely change over time. Besides cost, both assays offer advantages in different settings. The PCR-HRM approach is better suited for high throughput because the MIC-4 thermocycler can accommodate batches of 48 samples, compared to the Biomeme Franklin three 9 limit of 3 samples with 3 targets (maximum of nine tests per run). Biomeme’s lyophilized master mixes and probes are advantageous in truly field-forward situations, where cold storage facilities are unavailable, and where the operator may have limited technical experience. Based on findings from the present study, the cPCR assay using trans 1 and trans 2 primers for *C. burnetii* screening, was not an ideal platform to detect *C. burnetii* DNA in whole tick extracts. The differences in primer lengths across the PCR tests could influence the detection of *C. burnetii* DNA. The longer primer length in the cPCR compared to the shorter lengths in Biomeme qPCR and PCR-HRM might impact the sensitivity and specificity of each test. This variation is intrinsic to the nature of the primers and was a significant factor in our decision to compare these methods. Our results should be interpreted with this consideration in mind, and further studies could explore the impact of primer length on detection performance more comprehensively. Additional studies should evaluate the performance of cPCR using shorter primers such as those reported by Klee et al. (41). Ultimately, the choice of diagnostic test should be based on practical considerations that include the goals of the surveillance program as well as available resources (60).

Estimated specificity was high for all three tests, with the Sp central estimates ranging from 94% (PCR-HRM) to 98% (cPCR). Other reports of Sp for *C. burnetii* detection by Biomeme or PCR-HRM were not available for comparison. Test specificity describes the proportion of individuals without infection who are correctly identified as negative by a screening test. A high test specificity is especially important when there is low pathogen prevalence. Therefore, the *C. burnetii* PCR-HRM assay could be useful for surveillance in low prevalence or negative populations.

In this study*, C. burnetii* DNA was successfully detected in ticks collected from both wild animals and cattle, emphasizing the value of using tick samples for disease surveillance in areas where *C. burnetii* is endemic. Our model estimated that 16% of ticks collected tested positive for *C. burnetii* DNA, which is consistent with the endemic status of the pathogen in this area. Serological surveys in northern Kenya have reported seroprevalence rates of 10.3% in wild animals (30), 12.8–83.7% in livestock (19, 20, 28, 35) and 16.2–24.4% in humans (23, 35). It is unknown how accurately these estimates of *C. burnetii* in ticks translate into true infection prevalence due to the (inherently biased) opportunistic nature of sampling. In addition, positive tick samples may cluster by host species. Therefore, we exercise caution in interpreting our findings of *C. burnetii* in ticks as a true prevalence. It does, however, offer insight into the endemicity of *C. burnetti* in the ecosystem and the usefulness of ticks in revealing this burden.

Ticks can serve as indicators of *C. burnetii* presence in an ecosystem due to their involvement in the sylvatic transmission cycle (2, 14), however, their utility for disease surveillance is not well-established. Ndeereh et al. (30) reported the prevalence of *C. burnetii* in ticks collected from wild animals in Laikipia, Kenya, to be 0.54% (4/137 tick pools). In contrast, a study of ticks collected from ruminants in South Africa detected varying levels of zoonotic pathogens, including 7% positivity for *C. burnetii* DNA (61). This finding suggests that ticks may offer an advantageous and alternative sample type for surveillance of *C. burnetii*. They are widely distributed and can provide a non-invasive means of detecting pathogens, as shown in other studies (19, 29, 62–64). Validated methods to collect ticks from the environment already exist (65, 66) and they can facilitate widespread geographic coverage. This approach may provide insight on the *C. burnetii* status in a region without the need to handle individual animals, restrict sampling activities to culling/hunting seasons, or rely solely on opportunistic post-mortem examinations of wild animals.

Detection of *C. burnetii* in ticks may vary due infection prevalence in different hosts, sampled populations or environments, tick collection procedures, laboratory processing, and screening methods. Previously, studies have relied on cPCR for *C. burnetti* DNA detection in ticks, which the current study found lacking in sensitivity. Therefore, previous studies may have led to an underestimation and under recognition of the role of ticks in *C. burnetii* epidemiology. Further studies using larger numbers of ticks that represent multiple animal populations and incorporate more sensitive detection methods, such as the PCR-HRM assay, would improve estimates of true *C. burnetii* burden and could elucidate drivers of variability in *C. burnetii* DNA detection in ticks.

Since the primary goal of this study was to compare available detection platforms, Bayesian latent class analysis was used to estimate test performance of three *C. burnetii* PCR methods for detection in ticks. This analytic approach was selected since it does not depend on gold standard test results and has been increasingly used to evaluate and compare diagnostic tests (48). In the present study, a single population model was applied (48) and we assumed conditional independence of tests. This assumption was based on the three diagnostic assays using different primers and amplifying different segments of the IS*1111* transposase elements in the *C. burnetii* genome. Models that incorporate covariance (non-independence of assays) could be considered; however, in the present study, we did not have sufficient sample size or additional populations to fit covariance models based on recommended guidelines (48). Repeated studies with larger sample sizes, and multiple comparison populations, could address this limitation as well as reduce the variability in model estimates (67). As knowledge on these assays advance, the inclusion of prior knowledge (including the estimates published herein) into the Bayesian modeling approach will improve inferences.

We acknowledge that there were limitations in this study. First, the estimates of test performance did not incorporate screening of tick pools (usually 3–5 adult ticks or 10 nymphs per pool) (68), which could be a more economic option for screening (48, 69). Therefore, future studies should evaluate test performance using a pooled testing approach. Secondly, the primer target (IS*1111* insertion sequence) and length (290 bp) used in the Biomeme assay were known; however, the specific primer sequence was unavailable since it is proprietary. Therefore, confirmatory sequencing for *C. burnetii* could only be conducted using amplicons re-amplified by cPCR. A notable limitation was the variation in primer lengths among the PCR tests. While each test targeted different segments of the IS1111 transposase elements in the *C. burnetii* genome and was thus considered an independent detection method, the differences in primer lengths (687 bp for cPCR, 290 bp for Biomeme qPCR, and 150 bp for PCR-HRM) could introduce variability in test performance. Future research should aim to standardize primer lengths to minimize this variability and enhance the comparability of different PCR methods. Finally, the three assays were assessed using tick DNA samples, which are distinct from clinical samples in that they may contain *Coxiella*-like endosymbionts (2). These endosymbionts are solely found in ticks, where they enhance fitness, and are genetically related to *C. burnetii* (2). Consequently, they may be detected by certain PCR assays (based on primers) and could lead to biased estimates of *C. burnetii* abundance in an ecosystem (70). Future studies should determine whether the three assays perform differently when applied to clinical samples, where endosymbionts would not affect assay interpretation and infection burden can be more directly estimated.

## 5 Conclusion

The findings from this study demonstrate the value of using ticks from wildlife and cattle to detect the presence of *C. burnetii* in an ecosystem. A comparison of PCR assays showed that the PCR-HRM is a suitable screening test for *C. burnetii* using ticks; this test has good performance, is field-friendly, and delivers rapid results. While the PCR-HRM had higher Se compared to the Biomeme and cPCR, the Biomeme assay offers some advantages in certain settings. Findings from this study provide a first step to improve feasibility of noninvasive *C. burnetii* surveillance in ecosystems with human-wildlife-livestock interfaces. Future studies should evaluate these newer assays using multiple populations, different sample types, and similar BLCA methods.

## Data availability statement

All relevant data are contained within the article. The original contributions presented in the study are included in the article/Supplementary material, further inquiries can be directed to the corresponding author. The sequence data have been deposited in the European Nucleotide Archive (ENA) at EMBL-EBI under accession number PRJEB67399 (https://www.ebi.ac.uk/ena/browser/view/PRJEB67399).

## Ethics statement

The animal study was approved by Stellenbosch University Animal Use and Care (ACU-2023-27912) and Kenya’s National Commission for Science, Technology, and Innovation (NACOSTI/P/21/8054). The study was conducted in accordance with the local legislation and institutional requirements.

## Author contributions

MK: Software, Project administration, Writing – review & editing, Writing – original draft, Visualization, Methodology, Investigation, Formal analysis, Data curation, Conceptualization. CW: Writing – review & editing, Writing – original draft, Visualization, Supervision, Software, Methodology, Formal analysis, Data curation. WG: Validation, Writing – review & editing, Writing – original draft, Visualization, Supervision, Software, Methodology, Investigation, Formal analysis, Data curation, Conceptualization. MMu: Visualization, Validation, Investigation, Writing – review & editing, Resources, Methodology, Data curation. JV: Writing – review & editing, Visualization, Validation, Supervision, Software, Resources, Methodology, Investigation, Funding acquisition, Formal analysis, Data curation. DG: Writing – review & editing, Visualization, Validation, Software, Methodology, Investigation, Formal analysis, Data curation. RK: Writing – review & editing, Visualization, Validation, Software, Methodology, Investigation. MF: Writing – review & editing, Visualization, Validation, Supervision, Software, Resources, Methodology, Investigation, Formal analysis, Data curation. EF: Supervision, Writing – review & editing, Visualization, Validation, Software, Methodology, Investigation, Formal analysis, Data curation. DZ: Writing – review & editing, Visualization, Validation, Supervision, Resources, Project administration, Methodology, Investigation, Funding acquisition, Formal analysis, Conceptualization. Y-ML: Writing – review & editing, Visualization, Validation, Supervision, Software, Resources, Project administration, Methodology, Investigation, Funding acquisition, Data curation, Conceptualization. MMi: Writing – review & editing, Writing – original draft, Visualization, Validation, Supervision, Software, Resources, Project administration, Methodology, Investigation, Funding acquisition, Formal analysis, Data curation, Conceptualization.
