# Supplementary material for: Comparison of test performance of a conventional PCR and two field-friendly tests to detect Coxiella burnetii DNA in ticks using Bayesian latent class analysis
Source: Front Vet Sci. 2024 Jun 19;11:1396714. doi: 10.3389/fvets.2024.1396714 (PMC11220323; doi:10.3389/fvets.2024.1396714)
Supplement: Supplementary file 1 [file Table_1.DOCX]

***Supplementary Material for***

***Kamau et. al., 2024. Comparison o******f test performance of a conventional PCR and two field-friendly tests to detect Coxiella burnetii DNA in ticks using a Bayesian latent class analysis***

**Appendix S1: ADDITIONAL DATA AND SUMMARIZED RESULTS**

**Table S1:** A summary of tick DNA samples opportunistically collected from 17 wildlife species and cattle in wildlife conservancies in Kenya, between October 2011 and April 2019 and tested for *Coxiella burnetii* by conventional PCR, on the Biomeme *C. burnetii* platform, and a *C. burnetii* PCR HRM technique (n=179 ticks from 179 hosts).

|  |  |  | ***C. burnetii* results** | | |
| --- | --- | --- | --- | --- | --- |
| **Source host species** **(n individuals)** | **Tick genus (n)** | **n ticks tested***   **(subset with Biomeme, if different)** | **cPCR pos n**   **(% pos in host species)** | **Biomeme pos n**  **(% pos in host species)** | **PCR-HRM pos n**  **(% pos in host species)** |
| Black rhino (3) | *Rhipicephalus* (3) | 3 | 0 | 0 | 0 |
| Cattle (44) | *Rhipicephalus* (4)*;* Unk (40) | 44 (38) | 1 (2.6%) | 3 (6.8%) | 4 (9.1%) |
| Buffalo (36) | *Rhipicephalus* (21)*;* Unk (15) | 36 (35) | 2 (5.7%) | 8 (2.2%) | 8 (2.2%) |
| Cheetah (1) | Unk (1) | 1 | 0 | 0 | 0 |
| Eland (9) | Unk (9) | 9 | 0 | 0 | 1 (11.1%) |
| Elephant (17) | *Rhipicephalus* (7); Unk (10) | 17 | 1 (5.9%) | 0 | 2 (11.8%) |
| Grevy zebra (14) | *Rhipicephalus* (7); Unk (7) | 14 (13) | 2 (15.4%) | 1 (7.1%) | 4 (28.6%) |
| Giraffe (12) | *Rhipicephalus* (1); Unk (11) | 12 | 0 | 3 (25%) | 2 (16.7%) |
| Hartebeest (2) | *Rhipicephalus (2)* | 2 | 1 (50%) | 2 (100%) | 2 (100%) |
| Hyena (4) | *Rhipicephalus (*3); *Amblyomma (*1) | 4 | 0 | 0 | 1 (25%) |
| Impala (1) | *Rhipicephalus (1)* | 1 | 0 | 0 | 0 |
| Kudu (1) | Unk (1) | 1 | 0 | 0 | 0 |
| Leopard  (1) | *Rhipicephalus (1)* | 1 | 0 | 1 (100%) | 1 (100%) |
| Lion (13) | *Rhipicephalus (*6); *Amblyomma (*1); Unk (6) | 13 (12) | 1 (8.3%) | 1 (7.7%) | 3 (23.1%) |
| Plains zebra (7) | *Rhipicephalus* (2); *Amblyomma (1)*; Unk (4) | 7 | 1 (14.3%) | 1 (14.3%) | 3 (42.9%) |
| White rhino (4) | *Rhipicephalus* (1); Unk (3) | 4 | 0 | 0 | 0 |
| Wild dog (6) | *Rhipicephalus (*4); Unk (2) | 6 | 0 | 0 | 1 (16.7%) |
| Unknown/Not recorded (4) | *Rhipicephalus* (1); Unk (3) | 4 (3) | 0 | 1 (25%) | 0 |
| **Totals** | *Rhipicephalus* (64; 35.8% of 179);   *Amblyomma* (3; 1.7% of 179);   Unk (112; 62.6% of 179) | 179 (169) | 9   (5% of 179) | 21   (12.4% of 169) | 32   (17.9% of 179) |

cPCR=conventional PCR; pos=positive; Unk= Unknown spp.

*One tick from each individual animal was tested for *C. burnetii*.


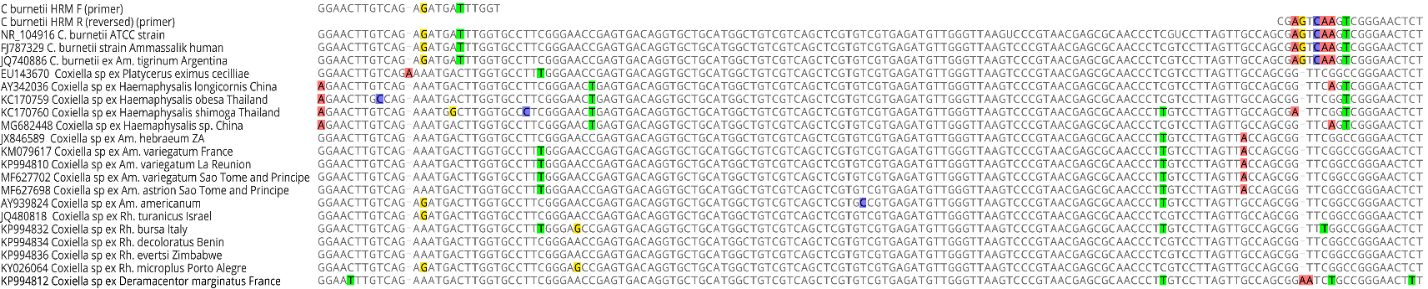


**Figure S1**: A figure showing the alignments of *Coxiella burnetii* and *Coxiella*-like endosymbionts nucleotide sequences available in the GenBank database and the 3' mismatches in the *C burnetii*_HRM-R primer.


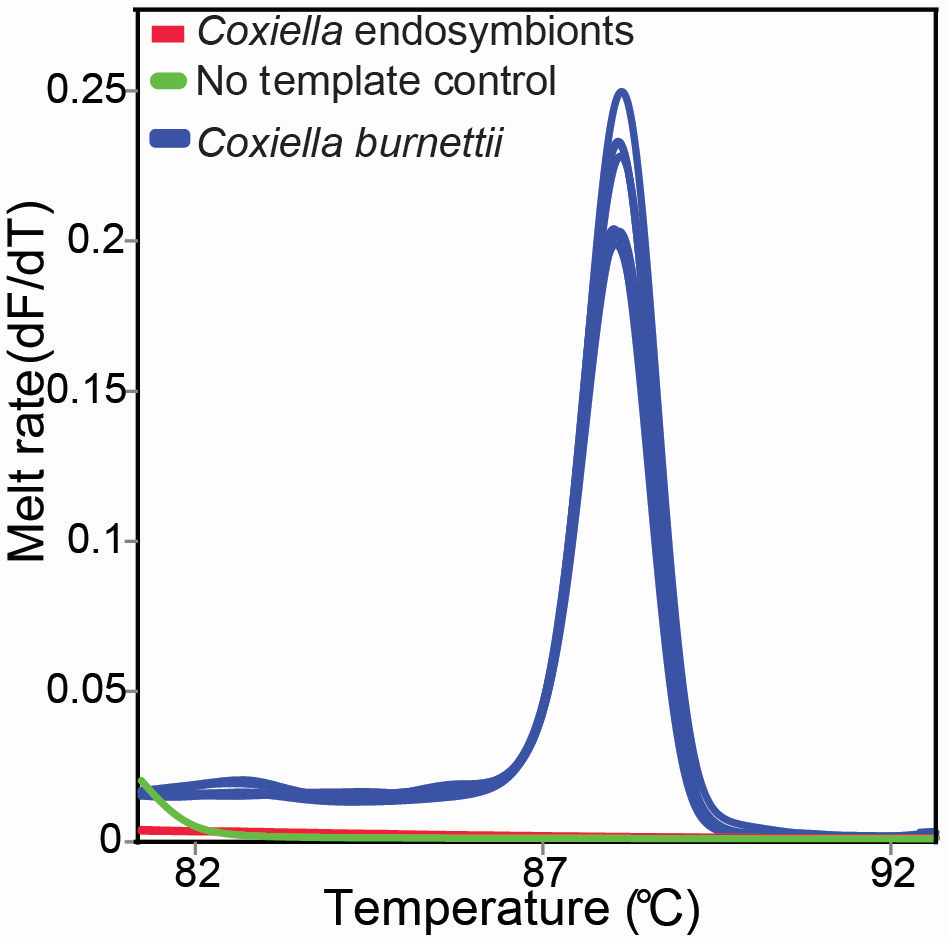


**Figure S2:** Figure showing the melt profiles of samples that had previously been confirmed to be positive for *Coxiella burnetii* and *Coxiella* endosymbionts as well as a no template control used to test the specificity of the *C_burnetii_*HRM-F and *C_burnetii*_HRM-R primers.

**Appendix S2: STARD-BLCM CHECKLIST**

**Table S1.** Checklist of items included for reporting the Bayesian Latent Class Model to aid in transparency, replicability, and integrity of the analysis. Checklist is from the Standards for the Reporting of Diagnostic Accuracy studies that use Bayesian Latent Class Models (STARD-BLCM), reported by Kostoulas et. al., 2017.

| Section and topic | Item | STARD BCLM | Section (section no.) |
| --- | --- | --- | --- |
| Title/  Keywords | 1 | Identification as a study of diagnostic accuracy, using at least one measure of accuracy (such as sensitivity, specificity, predictive values, or AUC) and Bayesian latent class models | Title; adjusted to best represent the purposes of the study |
| Abstract | 2 | Structured summary of study design, methods, results, and conclusions | Abstract |
| Introduction | 3 | Scientific and clinical background, including the intended use and clinical role of the tests under evaluation | Introduction (1) |
|  | 4 | Study objectives and hypotheses, such as estimation of diagnostic accuracy of the tests for a defined purpose through BLCM | Introduction (1) |
| Study design | 5 | Whether data collection was planned before the tests were performed (prospective study) or after (retrospective study) | Methods (2.1) |
| Participants | 6 | Eligibility criteria and description of the source population | Methods (2.1) |
|  | 7 | On what basis potentially eligible participants were identified (such as symptoms, results from previous tests, inclusion in registry) | Not applicable |
|  | 8 | Where and when potentially eligible participants were identified (setting, location, and dates) | Methods (2.1) |
|  | 9 | Whether participants formed a consecutive, random or convenience series | Methods (2.1) |
| Test methods | 10 | Description of the tests under evaluation, in  sufficient detail to allow replication, and/or cite references | Methods (2.2) |
|  | 11 | Rationale for choosing the tests under evaluation in relation to their purpose | Methods (2.2) |
|  | 12 | Rationale for test positivity cut-offs or result  categories of the tests under evaluation,  distinguishing pre-specified from exploratory | Methods (2.2) |
|  | 13 | Whether clinical information was available to the  performers or readers of the tests under evaluation | Not Applicable |
| Analysis | 14a | BLCM model for estimating measures of  diagnostic accuracy | Methods (2.3.1); Results (3; Table 3) |
|  | 14b | Definition and rationale of prior information  and sensitivity analysis | Methods (2.3.1) |
|  | 15 | How indeterminate results of the tests under  evaluation were handled | Not applicable; no indeterminant results in this study |
|  | 16 | How missing data of the tests under evaluation were handled | Methods (2.3.1) |
|  | 17 | Any analyses of variability in diagnostic accuracy,  distinguishing pre-specified from exploratory | Not applicable; not done in the present study |
|  | 18 | Intended sample size and how it was determined | Not applicable; samples size was determined opportunistically |
|  | 19 | Flow of participants, using a diagram | Not applicable |
|  | 20 | Baseline demographic and clinical characteristics of participants | Partially applicable as stated in Methods (2.3); tick hosts are reported in Appendix S1, Table S1 |
|  | 21 | Question not applicable per STARD BLCM standards | Not applicable |
|  | 22 | Time interval and any clinical interventions between the tests under evaluation | Not applicable |
| Test results | 23 | Cross tabulation of the tests’ results (or for continuous tests results their distribution by infection stage) | Table 2 |
|  | 24 | Estimates of diagnostic accuracy under alternative prior specification and their precision (such as 95% credible/probability intervals) | Not applicable; only non-informative priors were used (2.3.1) |
|  | 25 | Report any adverse events from performing the of the tests under evaluation | Not applicable |
| Discussion | 26 | Study limitations, including sources of potential bias, statistical uncertainty, and generalisability | Discussion (4) |
|  | 27 | Implications for practice, including the intended use and clinical role of the tests under evaluation in relevant settings (clinical, research, surveillance etc.) | Discussion (4) |
| Other information | 28 | Registration number and name of registry | Not applicable |
|  | 29 | Where the full study protocol can be accessed | Data availability (11) and included in manuscript |
|  | 30 | Sources of funding and other support; role of funders | Funding (8) |

**Appendix S3: R PROGRAMMING CODE**

This appendix shows the R programming code for a Bayesian latent class model evaluating three diagnostic tests for *Coxiella burnetti* in ticks. Ticks were collected from wildlife and cattle located in wildlife conservancies in Northern Kenya between 2011 and 2019 (model n = 169 tick samples). Evaluated tests included a conventional PCR (cPCR), a PCR High Resolution Melting (PCR HRM) technique for *C. burnetii* and using the Biomeme *C. burnetii* Go-strip qPCR assays and thermocycler (referred to herein as “Biomeme”).

#####################################################################################

# COMPARISON OF TEST PERFORMANCE OF A CONVENTIONAL PCR AND TWO FIELD-FRIENDLY TESTS TO

# DETECT COXIELLA BURNETII DNA IN TICKS USING BAYESIAN LATENT CLASS ANALYSIS.

# R programming code for Kamau et al. 2023

# Code derived and adapted from:

# Cheung, et al. Bayesian latent class analysis when the reference test is imperfect. *Rev Sci # Tech*. 2021 Jun;40(1):271-286. doi: 10.20506/rst.40.1.3224.

# Salgadu et al. Bayesian latent class analysis to estimate the optimal cut-off for the MiLA # ELISA for the detection of *Mycoplasma b*ovis antibodies in sera, accounting for repeated # measures. *Prev. Vet Med.* 2022 Aug; 205:105694. doi: 10.1016/j.prevetmed.2022.105694

###############################################################################################

#Estimating Diagnostic Sensitivity (Se) & Specificity (Sp) for 3 tests in 1 population

#####################################################################################

# Coxiella in ticks, 3 tests: Test 1=cPCR, Test 2=Biomeme, Test 3=PCR_HRM

# Assumptions:

# 1 population, all tests are independent.

#read dataset from working directory

coxiella <- read.csv("coxiellaData.csv")

# load required libraries

library(R2OpenBUGS) #for driving OpenBUGS from R

library(coda) #for reading and diagnostics of MCMC chains

library(mcmcplots) #for plotting

##########################################################

# Assign a flat prior distribution for all model parameters

#variables below are called later in the code

pi1.s1 <- 1; pi1.s2 <- 1

cPCR.se.s1 <- 1; cPCR.se.s2 <- 1

cPCR.sp.s1 <- 1; cPCR.sp.s2 <- 1

biomeme.se.s1 <- 1; biomeme.se.s2 <- 1

biomeme.sp.s1 <- 1; biomeme.sp.s2 <- 1

PCRhrm.se.s1 <- 1; PCRhrm.se.s2 <- 1

PCRhrm.sp.s1 <- 1; PCRhrm.sp.s2 <- 1

##########################################################

# Specify the model:

model=paste0("model{

#Multinomial Model for the Data

x1[1:2,1:2,1:2] ~ dmulti(p1[1:2,1:2,1:2], n1)

# 1=positive, 2=negative

# +++ All tests are positive

p1[1,1,1] <- (pi1* (Se1*Se2*Se3)) + ((1-pi1)* ((1-Sp1)*(1-Sp2)*(1-Sp3)))

#-++,+-+,++- All combinations of 1 test negative

p1[2,1,1] <- (pi1* ((1-Se1)*Se2*Se3)) + ((1-pi1)* (Sp1*(1-Sp2)*(1-Sp3)))

p1[1,2,1] <- (pi1* ((Se1*(1-Se2)*Se3))) + ((1-pi1)* ((1-Sp1)*Sp2*(1-Sp3)))

p1[1,1,2] <- (pi1* ((Se1*Se2*(1-Se3))) + ((1-pi1)* ((1-Sp1)*(1-Sp2)*Sp3))

# --+,-+-,+-- All combinations of 2 tests negative

p1[2,2,1] <- (pi1* ((1-Se1)*(1-Se2)*Se3)) + ((1-pi1)* (Sp1*Sp2*(1-Sp3)))

p1[2,1,2] <- (pi1* ((1-Se1)*Se2*(1-Se3))) + ((1-pi1)* (Sp1*(1-Sp2)*Sp3))

p1[1,2,2] <- (pi1* (Se1*(1-Se2)*(1-Se3))) + ((1-pi1)* ((1-Sp1)*Sp2*Sp3))

# --- All 3 tests are negative

p1[2,2,2] <- (pi1* ((1-Se1)*(1-Se2)*(1-Se3) ) + ((1-pi1)* (Sp1*Sp2*Sp3 ))

##############################################

# apply flat prior distributions to all parameters

############ Priors for main parameters

############ Assign a flat prior distribution (1,1) for all model parameters

pi1 ~ dbeta(",pi1.s1,", ",pi1.s2,")

Se1 ~ dbeta(",cPCR.se.s1,", ",cPCR.se.s2,")

Sp1 ~ dbeta(",cPCR.sp.s1,", ",cPCR.sp.s2,")

Se2 ~ dbeta(",biomeme.se.s1,", ",biomeme.se.s2,")

Sp2 ~ dbeta(",biomeme.sp.s1,", ",biomeme.sp.s2,")

Se3 ~ dbeta(",PCRhrm.se.s1,", ",PCRhrm.se.s2,")

Sp3 ~ dbeta(",PCRhrm.sp.s1,", ",PCRhrm.sp.s2,")

}")

#write to temporary text file

write.table(model, file="model.txt", quote=FALSE, sep="", row.names=FALSE, col.names=FALSE)

########

#Data

coxiella.dat <- na.omit(coxiella)

## organize variables as binary factors with 1 listed first, then zero

T1 <- as.factor(coxiella.dat$cPCR)

T1 <- factor(T1,levels=c(1,0))

T2 <- as.factor(coxiella.dat$biomeme)

T2 <- factor(T2,levels=c(1,0)) # in orig data, 1 = pos, 0 = neg

T3 <- coxiella.dat$PCRhrm

T3 <- factor(T3,levels=c(1,0)) # in orig data, 1 = pos based on cutoff identified from unexposed cohort of 20 goats

n1 <- length(T1)

x1 <- table(T1,T2,T3)

#set data inputs to BUGS

dat <- list("x1","n1")

#Set parameters desired to monitor

paras1<- c("Se1","Sp1","Se2","Sp2","Se3","Sp3","pi1")

paras_names<-c("cPCR Se", "cPCR Sp", "Biomeme Se", "Biomeme Sp", "PCR-HRM Se", "PCR-HRM Sp", "Proportion")

#Initialising values for 3 chains

inits1<-list(

list(Se1=0.50, Sp1=0.95, Se2=0.8, Sp2=0.95, Se3=0.85, Sp3=0.97, pi1=0.15),

list(Se1=0.45, Sp1=0.97, Se2=0.60, Sp2=0.98, Se3=0.7, Sp3=0.95, pi1=0.1),

list(Se1=0.40, Sp1=0.90, Se2=0.75, Sp2=0.95, Se3=0.8, Sp3=0.97, pi1=0.13)

)

##########################################################

### run model in OpenBUGS ###

niterations=30000

#final model

bug.out <- bugs(data=dat, inits=inits1, parameters.to.save=paras1, n.iter=niterations+1, n.burnin=0, n.thin=1, n.chains=3, model.file="model.txt", debug=T)

##########################################################

### model diagnostics and outputs ###

# preliminary output to check neffective and rhat

bug.out

# Diagnostics are available model output is converted into an

#MCMC object with this command:

bug.mcmc <- as.mcmc(bug.out)

# html files with trace, density, and autocorrelation plots

dir.name<-"mcmcout"

if(!dir.exists(dir.name)){dir.create(dir.name)}

mcmcplot(bug.out, title="Diagnostic plots",

filename = "_MCMCoutput",

dir = paste0(".//",dir.name,"//"),

extension = "html")

# Here, we consider convergence to be achieved after10000 iterations

# therfore, burn-in was set to so set to 10000

burnin <- 10000

##########################################################

# Final estimates

k<-seq(burnin+1,niterations,1)

# combine chains post-burnin

est<-data.frame(rbind(bug.out$sims.array[k,1,],bug.out$sims.array[k,2,]))

res<-t(apply(est,2,quantile,probs=c(0.5, 0.025, 0.975)))

round(res, 3)

res
